# Columnar cells necessary for motion responses of wide-field visual interneurons in *Drosophila*

**DOI:** 10.1007/s00359-012-0716-3

**Published:** 2012-03-13

**Authors:** Bettina Schnell, Shamprasad Varija Raghu, Aljoscha Nern, Alexander Borst

**Affiliations:** 1 Department of Systems and Computational Neurobiology, Max-Planck-Institute of Neurobiology, 82152 Martinsried, Germany; 2 Janelia Farm Research Campus, Howard Hughes Medical Institute, Ashburn, VA 20147 USA; 3 Present Address: Department of Biology, University of Washington, Box 351800, Kincaid Hall, Seattle, WA 98195 USA; 4 Present Address: Neuroscience Research Partnership, 138673 Singapore, Singapore

**Keywords:** Visual motion detection, Drosophila melanogaster, Lobula plate, Columnar cells, Patch-clamp recordings

## Abstract

Wide-field motion-sensitive neurons in the lobula plate (lobula plate tangential cells, LPTCs) of the fly have been studied for decades. However, it has never been conclusively shown which cells constitute their major presynaptic elements. LPTCs are supposed to be rendered directionally selective by integrating excitatory as well as inhibitory input from many local motion detectors. Based on their stratification in the different layers of the lobula plate, the columnar cells T4 and T5 are likely candidates to provide some of this input. To study their role in motion detection, we performed whole-cell recordings from LPTCs in *Drosophila* with T4 and T5 cells blocked using two different genetically encoded tools. In these flies, motion responses were abolished, while flicker responses largely remained. We thus demonstrate that T4 and T5 cells indeed represent those columnar cells that provide directionally selective motion information to LPTCs. Contrary to previous assumptions, flicker responses seem to be largely mediated by a third, independent pathway. This work thus represents a further step towards elucidating the complete motion detection circuitry of the fly.

## Introduction

**Fig. 1:**
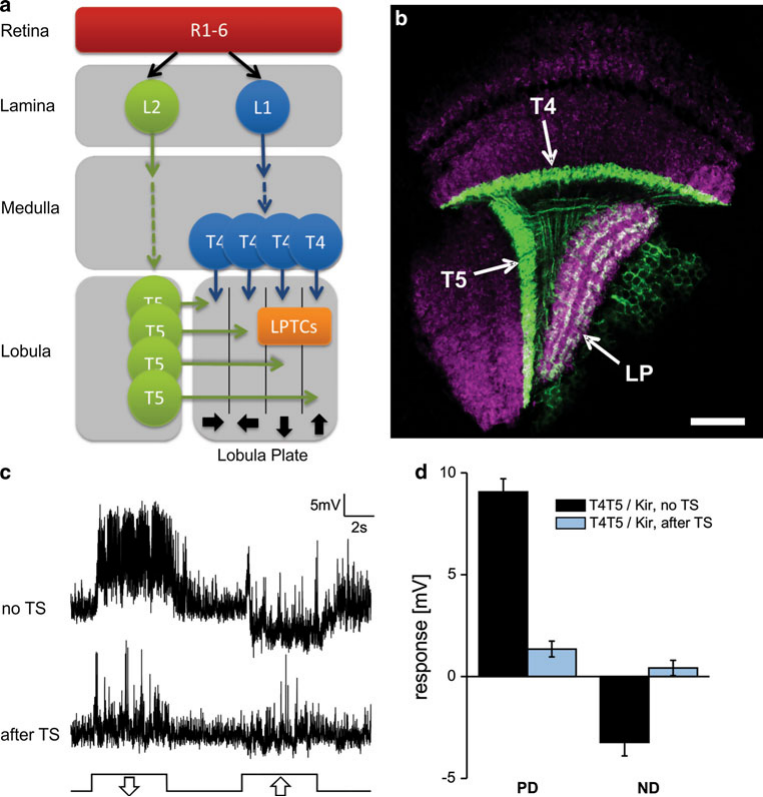
**a** Scheme of the optic neuropile depicting the two proposed pathways for motion detection leading from the retina to the lobula plate. **b** Single horizontal optical section of the optic lobe of a fly expressing Kir2.1-EGFP in T4 and T5 cells under control of the Gal4 driver line R42F06. LP = lobula plate, *scale bar* 20 μm. **c** Example responses of a frontal VS cell to downward (PD) and upward (ND) motion of a sine grating (temporal frequency = 1 Hz) of a control fly [top, no temperature shift (‘TS’)] and an experimental fly (bottom, after temperature shift). Both flies had the same genotype, but in experimental flies expression of Kir in T4 and T5 cells was induced by a temperature shift. In the experimental fly, the motion response is almost completely abolished. **d** Mean responses to PD and ND motion as shown in **c** for control flies (*n* = 4, 1 HS and 3 VS cells) not subjected to a temperature shift (‘TS’) and experimental flies (*n* = 7, 3 HS and 4 VS cells) after the shift. Motion responses are strongly reduced, yet a slight, but significant, difference between PD and ND motion remains (*p* = 0.008, one-tailed Wilcoxon Signed-Rank test). *Error bars* indicate SEM

Motion processing in the visual system of the fly has recently regained considerable attention due to the advances of genetic and physiological techniques in *Drosophila* (Borst 2009). These techniques promise mapping of the complete motion detection circuitry in the near future. Wide-field motion-sensitive neurons of the lobula plate, called ‘lobula plate tangential cells’ or ‘LPTCs’, have been studied for long. They respond to motion in a directionally selective way. Among them, cells of the vertical (‘VS’) and horizontal system (‘HS’) are the major output neurons. In *Drosophila*, there are at least six VS cells responding primarily to vertical motion (Joesch et al. 2008; Maimon et al. 2010) and three HS cells responding preferentially to horizontal motion (Schnell et al. 2010; Chiappe et al. 2010), which occupy the outer- and innermost layers of the lobula plate, respectively. They are thought to integrate the outputs of hundreds of local motion-sensitive elements on their large dendrites. According to a well-established algorithmic model, the so-called Reichardt detector, these elements extract directional information from the changing retinal images by correlating the luminance information from adjacent photoreceptors after one of them has been delayed by a low-pass filter (Reichardt 1987). Combining genetic blockage of two cell types postsynaptic to photoreceptors in the lamina, L1 and L2, with recordings from LPTCs in *Drosophila* led to a refined version of the model involving separate channels for detecting moving brightness increments and decrements (Joesch et al. 2010; Clark et al. 2011; Eichner et al. 2011,). However, it remained largely unknown as to which cell types intervene between L1/L2 and LPTCs and, thus, constitute the circuit for elementary motion detection. Based on anatomical studies, T4 and T5 cells are assumed to be presynaptic to LPTCs and to provide input from the L1 and L2 pathways, respectively (Bausenwein et al. 1992). The dendrites of T4 occupy the most proximal layer of the medulla, while dendrites of T5 cells are located in the posterior layer of the lobula (Fischbach and Dittrich 1989). Both cell types come in four different variants each projecting to one out of four different layers in the lobula plate, each of which is dedicated to the processing of motion in one out of four different directions (Buchner et al. 1984) (Fig. 1a). A few rare recordings from T4 and T5 cells in blowflies suggest that at least T5 is directionally selective (Douglass and Strausfeld 1995; Douglass and Strausfeld 1996). Furthermore, a chemical synapse between a T4 cell and an LPTC has been described in an EM study (Strausfeld and Lee 1991). Thus, while a lot of circumstantial evidence makes T4 and T5 cells the prime candidates for directional input to the LPTCs, this has never been demonstrated directly. Furthermore, as LPTCs receive excitatory and inhibitory input (Raghu et al. 2007; Raghu et al. 2009) and also respond to overall changes in luminance, the question is whether T4 and T5 provide all of that input or whether other columnar cells participate as well. To study that question, we generated flies that express a neuronal blocker in T4 and T5 cells using the Gal4-UAS system. By performing whole-cell recordings from VS and HS cells in these flies, we show that T4 and T5 are necessary for motion responses in these cells.

## Materials and methods

### Flies

Flies were raised on standard cornmeal agar medium at a 12 h light/12 h dark cycle, 25°C and 60% humidity. We used female experimental flies, 1 day after eclosion. For blocking experiments, two effector strains were used, one carrying a single insertion of UAS-*Kir2.1*-*EGFP* on the second and *tub*-*Gal80* ^*ts*^ on the third chromosome, the other one carrying the *white* gene and multiple insertions of UAS-*shi* ^*ts*^ on the third chromosome. Heterozygous control and experimental flies were obtained by crossing the respective Gal4-driver and UAS-effector strains. For experiments with Kir, experimental flies were initially raised at 25°C and shifted to 31°C, 2–5 days prior to hatching to inactivate Gal80^ts^. Control flies were kept at 25°C throughout development. For experiments with Shi^ts^, control and experimental flies had identical genotype and were raised at 25°C (permissive temperature). Experimental flies were shifted for 1 h to 37°C (restrictive temperature) directly before the experiment and recorded at room temperature within 1 h after the temperature shift. No recovery of the block was detected within the time of the recording. As a control for effects of the temperature treatment, flies with UAS-*shi* ^*ts*^ were crossed with wild type flies (Canton S, CS’) and subjected to the same temperature regime as experimental flies. The Gal4 driver line R42F06, leading to selective expression in T4 and T5 cells, is from the Janelia farm collection and was generated as described previously (Pfeiffer et al. 2008). Briefly, the 3,990-bp enhancer fragment driving Gal4 expression was amplified with PCR from the non-coding region flanking the gene CG9102 or *bab2* (chromosome 3L: 1147066 to 1151056, primers: cggctgatccaacaaaggatgcacc, ctcagtgtagccgcaccttgttcct) and inversely cloned into the pBPGUw vector.

### Preparation

Flies were anaesthetized on ice and waxed on a Plexiglas holder using bee wax. The dissection of the fly cuticle and exposure of the lobula plate were performed as described previously (Joesch et al. 2008). The neurolemma was either digested by mild Protease treatment (Protease XIV, P-5147, Sigma Aldrich; 2 mg/ml, max 4 min) as in Schnell et al. (2010) or, for the experiments on Shi^ts^ flies, by Collagenase treatment (Maimon et al. 2010). In the latter case, a cleaning electrode was filled with Collagenase solution (0.5 mg/ml, Collagenase IV, Worthington) and was moved from side to side above the LPTC somata while applying pressure until the neurolemma disrupted and the somata became visible. In some cases, remains of the neurolemma and glia cells were mechanically removed using a recording electrode.

### Whole-cell recording

VS and HS cell somata covered by Ringer’s solution were approached with a recording glass pipette (7–10 MΩ) filled with a red fluorescent dye (intracellular solution as in Joesch et al. 2008). Recordings were established under high-contrast optics using a 40× water immersion objective (LumplanF, Olympus), a Zeiss Microscope (Axiotech vario 100, Zeiss) and illumination (100 W fluorescence lamp, heat mirror, neutral density filter OD 0.3; all from Zeiss). To enhance tissue contrast, we used two polarization filters, one located as an excitation filter and the other as an emission filter, with slight deviation on their polarization plane. For eye protection, we additionally used a 420-nm LP filter on the light path. After the recording cells were filled with intracellular solution by applying negative current of about 0.5 nA for about 5 min. Cells were identified by eye inspection based on their dendritic arborizations. Only recordings from VS cells 1-4 and HSN, HSE, and HSS were used for this study.

### Immunohistochemistry and confocal microscopy

Female flies were dissected after 3 days on restrictive temperature. Their brains were removed and fixed in 4% paraformaldehyde for 30 min at room temperature. Subsequently, the brains were washed for 45–60 min in PBT (phosphate buffered saline (pH 7.2) including 1% Triton X-100). For antibody staining, the samples were further incubated in PBT including 2% normal goat serum (Sigma Aldrich, G9023) and primary antibodies (1:200, overnight at 4°C). Antibodies were removed by several washing steps (5 × 20 min in PBT) and secondary antibodies were added (1:200, overnight at 4°C). A 5 × 20 min washing protocol (PBT) was followed by final washing steps in PBS (5 × 20 min). The following primary and secondary antibodies were used in the present study: Alexa Fluor 488 rabbit anti-GFP-IgG (A-21311, Molecular Probes), mouse anti-Dlg (Developmental Studies Hybridoma Bank, University of Iowa, Iowa City) and mouse Alexa Fluor 568 (A-11004, Molecular Probes). The stained brains were mounted in Vectashield (Vector Laboratories, Burlingame). Serial optical sections were taken at 0.5-μm intervals with 1,024 × 1,024 pixel resolution using a confocal microscope (LEICA SP5) and an oil-immersion 63× (n.a. = 1.4) Plan-Apochromat objective. The size, contrast and brightness of the resulting images were adjusted using Image J (NIH, USA) software.

### Visual stimulation and data analysis

A custom-built cylindrical LED arena covered ~170° (1.4° resolution) of the horizontal and ~100° of the vertical visual field of the fly, allowing refresh rates of up to 600 Hz with 16 intensity levels. The spectral peak of the LEDs was at 568 nm and the luminance range of the stimuli was between 0.5 and 80 cdm^−2^ (for further details see Schnell et al. 2010). To study large-field motion responses, sine gratings of two different orientations, horizontal and vertical (spatial wavelength: 42.5° for the horizontal and 45° for the vertical patterns, contrast = 100%) moving in four different directions at a temporal frequency of 1 Hz were presented. PD and ND responses were calculated as the mean during the 5 s stimulus periods minus the baseline response (calculated as the mean during 500 ms before stimulus onset). For flicker stimuli, the whole arena was switched to maximal luminance for half a second and off again (contrast = 100%). Flicker stimuli were always presented at the beginning of the experiment to assure a comparable state of light adaptation. Peak responses were calculated as the maximal value within 100 ms after the stimulus minus the baseline (average potential during 100 ms before the stimulus).

## Results

To study the role of T4 and T5 in motion processing, we used the Gal4-UAS system to block their function while recording from a subset of LPTCs in *Drosophila*, i.e. VS and HS cells. We employed a Gal4 line that specifically labels T4 and T5 cells.

### Expression of Kir2.1-EGFP in T4 and T5

In a first set of experiments, we used this line to drive expression of the inward rectifying potassium channel Kir2.1 (in short: Kir) tagged with enhanced green fluorescent protein (‘EGFP’) (Baines et al. 2001). Kir is supposed to inactivate cells by hyperpolarization and shunting inhibition (Johns et al. 1999). The tagging with EGFP allows for visualizing its expression pattern in flies of the same genotype as used for the physiological experiments (see below). To induce expression of the channel in later stages of development, flies also contained the gene for a temperature sensitive Gal80, which inhibits Gal4 at the permissive temperature (Thum et al. 2006). Experimental flies were shifted to the restrictive temperature of 31°C for at least 2 days prior to hatching to inactivate Gal80 and induce expression of Kir. Control flies were not subjected to this temperature shift and showed no visible fluorescent labeling. After the temperature shift, however, Kir-EGFP was strongly expressed in the layers of the medulla and lobula, which contain the dendrites of T4 and T5 cells, respectively, in all four layers of the lobula plate as well as in the soma layer posterior to it (Fig. 1b). As there are no other cells known from Golgi studies that arborize in the specified layers (Fischbach and Dittrich 1989), we conclude that the cell types labeled are indeed T4 and T5. No other cells in the optic lobes showed expression of Kir-EGFP, thus demonstrating the specificity of this approach.

We performed whole-cell patch-clamp recordings from the cell bodies of VS cells 1-4 and HS cells while presenting a sine grating moving in the preferred (PD) or null direction (ND) of the cell (downward and upward for VS cells, front-to-back and back-to-front for HS cells). Control flies exhibited directionally selective motion responses, i.e., they depolarized in response to PD and hyperpolarized in response to ND motion (Fig. 1c). In contrast, directionally selective motion responses in VS as well as HS cells were almost completely abolished in flies expressing Kir in T4 and T5 cells (Fig. 1c, d). This holds true for depolarizing and hyperpolarizing responses. However, cells still responded strongly to changes in overall luminance (‘flicker responses’, data not shown, but see below).

### Expression of Shi^ts^ in T4 and T5

**Fig. 2:**
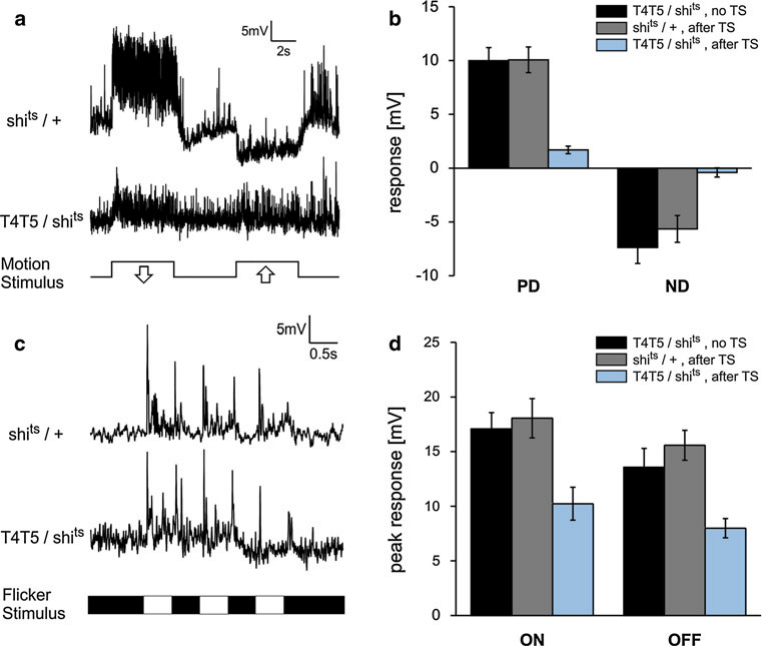
**a** Example responses of a frontal VS cell to downward (PD) and upward (ND) motion of a sine grating (temporal frequency = 1 Hz) of a control fly heterozygous for UAS-*shi* ^*ts*^ and an experimental fly expressing Shi^ts^ in T4 and T5 after 1 h at 37°C. In the experimental fly, the motion response is almost completely abolished. **b** Mean responses to PD and ND motion as shown in **a** for control flies carrying UAS-*shi* ^*ts*^ and the Gal4 driver, but not subjected to a temperature shift (TS) (*n* = 6, 1 HS and 5 VS cells), heterozygous *shi* ^*ts*^ flies without a Gal4 driver after the TS (*n* = 6, 1 HS and 5 VS cells) and experimental flies with Shibire expressed in T4 and T5 after the TS (*n* = 12, 3 HS and 9 VS cells). Motion responses are strongly reduced in experimental flies. Error bars indicate SEM. **c** Flicker response of a control and an experimental fly as in **a** in response to three consecutive light-on and off-stimuli (temporal frequency = 1 Hz). **d** Mean peak responses to the first on and off stimulus as in **c** for control (*n* = 6) and experimental flies (*n* = 12)

As another way of blocking T4 and T5 cells, we used the transgene UAS-*shi* ^*ts*^ (in short: shi^ts^). Shi^ts^ blocks chemical synapses by inhibiting endocytosis of synaptic vesicles at the restrictive temperature. For inducing the effect, we subjected adult flies to a temperature of 37°C for 1 h prior to the experiment, a regime that was previously shown to result in a block lasting for over 1 h after shifting flies back to room temperature (Joesch et al. 2010). All recordings were performed within this 1 h. As controls, we used flies that either had the same genotype but were not subjected to the temperature shift, or flies that lacked the Gal4-driver but underwent the same temperature protocol as the experimental flies did. As with Kir, induction of Shi^ts^ in T4 and T5 cells almost completely abolished motion responses in all cells recorded for all directions of motion. In contrast, LPTCs from control flies responded normally (Fig. 2a, b). A slight but significant difference between PD and ND responses is still apparent in the experimental flies (*p* = 0.001, one-tailed Wilcoxon Signed-Rank test). Nevertheless, T4 and T5 cells are clearly the key components providing motion input to LPTCs.

HS and VS cells do not only respond to motion stimuli but also transiently depolarize in response to sudden luminance changes of either polarity (‘flicker response’; Fig. 2c). Flicker responses in control flies reached high peak amplitudes, but varied more strongly than motion responses across individuals and trials (Fig. 2c). Experimental flies with T4 and T5 cells blocked still exhibited strong flicker responses to brightness increments and decrements, whose mean peak amplitudes were only reduced to about 60% of those from control flies (Fig. 2c, d). Since data for flicker and motion stimuli were obtained from the same flies, flicker responses persist despite motion responses being abolished.

## Discussion

Directionally selective LPTCs in *Drosophila* are assumed to integrate excitatory and inhibitory input from an array of local motion detectors. Based on recent evidence, motion information is, in addition, split into separate channels dedicated to moving brightness increments and decrements for each direction (Joesch et al. 2010; Eichner et al. 2011; Clark et al. 2011). The question thus arises as to which columnar cells provide all of that input to LPTCs. Based on their anatomy, T4 and T5 cells seemed likely candidates as they come in different variants projecting to different layers of the lobula plate. A synapse between a T4 and an HS cell could indeed be revealed in an EM study (Strausfeld and Lee 1991). However, there are other medulla cells that project to the lobula plate too (Fischbach and Dittrich 1989), so it remained unclear whether T4 and T5 provide the only columnar input to LPTCs or whether other cells contribute some of the input as mentioned above.

We studied this question by blocking T4 and T5 using two different genetic tools while recording from LPTCs in *Drosophila*. Motion responses of VS and HS cells were almost completely abolished in flies, in which either activity or synaptic transmission in T4 and T5 cells was blocked using Kir or Shi^ts^, respectively (Figs. 1d, 2b). As Kir was tagged with EGFP, we could confirm that its expression was confined to T4 and T5 cells in the optic lobes in flies of the same genotype as those used for recordings. The effect of Shi^ts^, on the other hand, can be induced on a shorter time scale thus preventing any side effects during development. As both tools lead to the same result, we are confident that lacking motion responses can be attributed to a functional block of T4 and T5 cells. However, a small difference in response to PD and ND motion was still apparent. Whether this remaining response is due to an incomplete block of T4 and T5 cells or whether there are other cells that provide an additional directionally selective input to VS and HS cells remains to be analyzed. The fact that depolarizing and hyperpolarizing responses in VS as well as HS cells were affected demonstrates that the processing of all four directions of motion relies on T4 and T5.

In contrast to motion stimuli, responses to flicker were only slightly reduced. This is surprising insofar as flicker responses were previously assumed to result from an imbalance between the excitatory and inhibitory motion detectors providing input to LPTCs (Egelhaaf et al. 1989), leading to a net depolarization when both detectors are activated by spatially uniform luminance changes. However, as both the excitatory and the inhibitory motion inputs are provided by T4 and T5 cells, flicker responses should be abolished as well in flies with these cells blocked. Consequently, a large part of the flicker responses seems to be mediated by a third, yet unknown input pathway. This finding offers an alternative explanation for the surprisingly strong flicker component in responses to apparent motion stimuli, where two brightness steps are sequentially presented at neighboring positions (Egelhaaf and Borst 1992; Eichner et al. 2011; Tuthill et al. 2011). However, the further conclusions drawn in these studies remain unaffected by our findings since the flicker responses were eliminated in the evaluation process by either subtracting them explicitly (Egelhaaf and Borst 1992) or subtracting the response to the ND sequence from the one to the PD sequence (Eichner et al. 2011). Concerning the function of a separate flicker pathway, it was shown previously that flies react to pure flicker stimuli (McCann and MacGinitie 1965; Pick 1974; Wehrhahn 1981) and flickering bars were also claimed to attract the visual attention of flies (Sareen et al. 2011). Whether LPTCs are involved in any of these responses remains to be studied.

Our findings mark another important step in the search for those columnar cell types that compute directionally selective motion information as postulated by the Reichardt detector. Based on anatomical studies, T4 and T5 were proposed to be part of two largely independent pathways leading from the photoreceptors to the LPTCs: The first one via L1, Mi1 and T4, the second one via L2, Tm1 and T5 (Bausenwein and Fischbach 1992; Bausenwein et al. 1992). These pathways seem to be largely conserved across fly species (Buschbeck and Strausfeld 1996). In *Drosophila*, L1 and L2 have already been shown to be key players for motion detection at the level of the lamina (Rister et al. 2007) giving rise to two parallel pathways dedicated to the processing of brightness increments and decrements, respectively (Joesch et al. 2010; Reiff et al. 2010; Clark et al. 2011; Eichner et al. 2011). A recent study also confirmed synaptic connections between L2 and the transmedullary cells Tm1 and Tm2 (Takemura et al. 2011). We now establish T4 and T5 as essential components of these pathways. Thus, about 100 years after these cells have first been described anatomically (Cajal and Sanchez 1915), we finally confirmed their major function in motion detection in the fly.

## Abbreviations

EGFP: Enhanced green fluorescent protein
HS: Horizontal system
Kir: Potassium inward rectifier
LPTC: Lobula plate tangential cell
ND: Null direction
PD: Preferred direction
VS: Vertical system
